# Point-of-Care and Label-Free Detection of Porcine Reproductive and Respiratory Syndrome and Swine Influenza Viruses Using a Microfluidic Device with Photonic Integrated Circuits

**DOI:** 10.3390/v14050988

**Published:** 2022-05-07

**Authors:** Georgios Manessis, Maciej Frant, Grzegorz Wozniakowski, Lapo Nannucci, Martina Benedetti, Lilla Denes, Balka Gyula, Athanasios I. Gelasakis, Clare Squires, Sara Recuero, Carlos Sanchez, Amadeu Griol, Alessandro Giusti, Ioannis Bossis

**Affiliations:** 1 Laboratory of Anatomy and Physiology of Farm Animals, Department of Animal Science, Agricultural University of Athens (AUA), Iera Odos 75 Str., 11855 Athens, Greece; gmanesis@aua.gr (G.M.); gelasakis@aua.gr (A.I.G.); 2 Department of Swine Diseases, National Veterinary Research Institute, Partyzantów Avenue 57, 24-100 Puławy, Poland; maciej.frant@piwet.pulawy.pl (M.F.); grzegorz.wozniakowski@umk.pl (G.W.); 3 Department of Diagnostics and Clinical Sciences, Faculty of Biological and Veterinary Sciences, Nicolas Copernicus University in Torun, Lwowska 1, 87-100 Torun, Poland; 4 Dipartimento di Scienze e Tecnologie Agrarie Alimentari Ambientali e Forestali, Università Degli Studi di Firenze, Piazzale delle Cascine 18, 50144 Florence, Italy; lapo.nannucci@unifi.it; 5 Istituto Zooprofilattico Sperimentale del Lazio e della Toscana ‘M. Aleandri’, Via Castelpulci 43, 50018 Florence, Italy; martina.benedetti@izslt.it; 6 Department of Pathology, University of Veterinary Medicine Budapest, Istvan Str. 2, 1078 Budapest, Hungary; denes.lilla@univet.hu (L.D.); balka.gyula@univet.hu (B.G.); 7 Laboratory of Animal Husbandry, Department of Animal Production, School of Agriculture, Faculty of Agriculture, Forestry and Natural Environment, Aristotle University of Thessaloniki, 54124 Thessaloniki, Greece; squires@vet.auth.gr; 8 Lumensia Sensors S.L., Camino de Vera, s/n, K-Access, Building 8F 3th-Floor, 46022 Valencia, Spain; srecuero@lumensia.com (S.R.); csanchez@lumensia.com (C.S.); 9 Nanophotonics Technology Center, Universitat Politècnica de València, Camino de Vera s/n Building 8F, 46022 Valencia, Spain; agriol@upvnet.upv.es; 10 Cyprus Research and Innovation Centre Ltd. (CyRIC), 28th Octovriou Ave 72, Off. 301, Engomi, Nicosia 2414, Cyprus; alessandro@cyric.eu

**Keywords:** point of care, diagnostics, photonic integrated circuits, microfluidics, porcine reproductive and respiratory syndrome virus, swine influenza A virus, oral fluids, validation, sensitivity, specificity, diagnostic odds ratio

## Abstract

Swine viral diseases challenge the sector’s sustainability by affecting productivity and the health and welfare of the animals. The lack of antiviral drugs and/or effective vaccines renders early and reliable diagnosis the basis of viral disease management, underlining the importance of point-of-care (POC) diagnostics. A novel POC diagnostic device utilizing photonic integrated circuits (PICs), microfluidics, and information and communication technologies for the detection of porcine reproductive and respiratory syndrome virus (PRRSV) and swine influenza A (SIV) was validated using spiked and clinical oral fluid samples. Metrics including sensitivity, specificity, accuracy, precision, positive likelihood ratio (PLR), negative likelihood ratio (NLR), and diagnostic odds ratio (DOR) were calculated to assess the performance of the device. For PRRSV, the device achieved a sensitivity of 83.5%, specificity of 77.8%, and DOR values of 17.66, whereas the values for SIV were 81.8%, 82.2%, and 20.81, respectively. The POC device and PICs can be used for the detection of PRRSV and SIV in the field, paving the way for the introduction of novel technologies in the field of animal POC diagnostics to further optimize livestock biosecurity.

## 1. Introduction

Animal production plays an important role in food security by providing products of high nutritional value and safeguarding the livelihoods of millions of people around the world. The swine industry is a prominent sector in animal production as it accounts for approximately 35% of total meat production [1,2]. Modern animal production is characterized by the intensification of farming systems on a global scale. Intensive farming systems are usually outlined by high inputs and increased stocking density to reduce production costs. However, increased stocking density can jeopardize animal health by facilitating pathogen transmission [3]. At the same time, transboundary infectious diseases are emerging due to globalized trade networks and scarce surveillance programs, further increasing the risk of disease outbreaks [4,5]. As the recent experience with the African swine fever virus has shown, viral diseases have the potential to devastate the swine sector due to (i) their transmission dynamics; (ii) the limited or non-existent treatment options; (iii) vaccine availability and efficiency; and (iv) the limited available preventive measures that are mainly based on hygiene and biosecurity [6,7,8,9,10].

Among the viral diseases, porcine reproductive and respiratory syndrome virus (PRRSV-1 and 2), comprising two species *Betaarterivirus suid* 1 and 2 (formerly known as European Type 1 and North American Type 2), and swine influenza A virus (SIV) are of great importance due to their economic impact and in the case of SIV, due to its zooanthroponotic potential. PRRSV is an enveloped, positive-strand RNA virus that belongs to the *Arteriviridae* family. Genomic RNA is approximately 15 kb in length and contains eight open reading frames (ORFs). About 80% of the genome consists of ORFs 1a and 1b, which encode the RNA replicase [11]. ORFs 2 to 5 encode the structural glycoproteins GP2 to GP5. ORF6 and ORF7 encode the structural membrane protein M and the nucleocapsid protein N, respectively [12]. The GP5 protein is the most variable structural protein between the North American and European isolates, with only 51–55% amino acid identity. In contrast, the M protein is the most conserved structural protein, reaching 78–81% amino acid identity. The N protein constitutes 20–40% of the PRRSV virion and is an immunodominant protein. Several N protein B-cell epitopes are conserved in both the European and North American isolates [13]. Therefore, antibodies that recognize the N protein appear to be suitable for diagnostic tests [12].

Prevailing clinical symptoms of PRRSV infection are respiratory distress and poor growth in nursing, growing, and finishing pigs as well as reproductive failure in pregnant sows including mummified, stillborn, and aborted fetuses [14]. Apart from the acute clinical manifestation of the disease, PRRSV can lead to life-long subclinical infections and is characterized by persistence at the herd level [15]. Outbreaks at a global scale are sustained by emerging and re-emerging strains due to mutations and viral recombinations [14] and cost around USD 664 million annually, in the USA alone [16]. Control of the disease at the farm level is based on hygiene and biosecurity as well as management practices such as pig flow, gilt acclimation, and vaccination [17]. Elimination of PRRSV at the farm level is accomplished by the culling of animals with severe symptoms and long-term herd closure (no importation of foreign animals) to achieve herd immunity; however, re-introduction of the virus is common despite the applied biosecurity measures [17]. Vaccination, elimination methods (test and removal, whole herd depopulation and repopulation, herd closure and rollover), and reducing persistence as well as intercepting the direct (infected pigs and contaminated semen) and indirect (fomites, vehicles, insects, and aerosols) transmission routes are key measures to mitigate PRRSV in farms [17,18]. It is worth mentioning that successful implementation of the above-mentioned measures would be facilitated by reliable and inexpensive diagnostics.

Influenza A viruses are enveloped, 80–120 nm in size, contain eight segments of negative sense single-stranded RNA and belong to the *Orthomyxoviridae* family [19]. The eight RNA segments vary in length (890–2341 nucleotides) and encode 10 and in some cases up to 12 proteins [20]. Segment 7 (Matrix, M) and segment 8 (Nonstructural, NS) encode two proteins, M1/M2 and NS1/NS2, respectively. The RNA segments are bound and protected by the viral nucleoprotein (NP). The RNA, the viral polymerase complex, and the NP form a ribonucleoprotein (RNP) complex. SIV is typed based on the combination of two surface glycoproteins, haemagglutinin (HA) and neuraminidase (NA) [20]. Currently 18 HAs and 11 NAs have been identified.

HA of avian origin binds to the N-acetylneuraminic acid–2,3-galactose linkage and HA of mammalian origin binds to the N-acetylneuraminic acid–2,6-galactose linkage of sialyloligosaccharides. Swine epithelial cells express both types of sialic acid linkages, enabling the co-infection of viruses of both avian and human origin, thus mediating virus reassortment [21]. Influenza viruses, previously adapted to swine, can combine and exchange genes with viruses of both human and avian origin in cases of co-infection, a phenomenon also known as antigenic shift, producing triple reassortments such as the 2009 H1N1 pandemic virus [21]. SIV RNA is also highly susceptible to point mutations. The accumulation of point mutations, substitutions, deletions, and insertions in the HA and NA encoding region of the RNA alters the antigenic properties of HA and NA, causing antigenic drifts. Both antigenic shift and antigenic drift commonly occur in SIV due to the fragmented nature of RNA, giving rise to novel reassortants/subtypes and novel strains [22]. This constant evolution of swine influenza A viruses is the cause of annual epizootics. SIV outbreaks are characterized by sudden onset of disease, high morbidity approaching 100%, and low mortality, typically less than 1%. The incubation period is between 1–3 days and the clinical manifestation includes fever, inactivity, decreased food intake, respiratory distress, coughing, sneezing, conjunctivitis, and nasal discharge, although the severity of the disease can be affected by the viral strain [20]. Animals recover 4–7 days after the onset of the disease. Occasionally, SIV reassortants can break the species barrier and infect humans. Control of SIV in swine farms is based on hygiene, partial depopulation, segregation of weaned piglets, and all-in/all-out systems. Vaccination is known to reduce the incidence and the severity of the disease, however, SIV vaccines are not constantly renewed due to cost and do not consistently provide complete protection. Immunization against both H1 and H3 subtypes should be preferred for the effective control of the disease in European pig herds [23].

The lack of effective antiviral drugs for PRRSV and SIV in animal production has established the prevention and disease control measures as the primary tools to control spread [24]. Thus, reliable and early diagnosis is critical for the implementation of targeted disease countermeasures. Prevailing diagnostic assays for the detection of swine viral diseases include some form of PCR, immunoassays, or less frequently, cell culture, hence requiring centralized laboratories, trained personnel, and significant delays from sampling to diagnosis [25]. As a result, the time between the clinical manifestation of viral diseases and laboratory confirmation may span from days to weeks, and often diagnosis does not coincide with the progress of the disease [26].

Point-of-care (POC) devices and tests are used on site to provide clinical information on health challenging conditions or diagnose diseases [27]. An ideal POC device should be user-friendly, portable, low-cost, efficient, able to operate with small volumes of complex samples, require minimal handling, enable multiplexing, monitor the health status, and be able to share information on site [28]. The most common forms of POC tests are dipstick, strip tests, and lateral flow assays, however, these tests may suffer from low sensitivity and relatively high detection limits [29]. Innovations such as micro- and nano-fabrication, information and communication technologies, photonics, microfluidics, and advanced materials have been exploited to improve the current POC devices and tests in terms of performance and expansion of the panel of measured markers, leading to the introduction of lab-on-a-chip (LOC) devices [30,31]. Up to the present, conventional diagnostic techniques such as PCR, LAMP, and ELISA have been translated to LOC devices [32,33,34]. Despite these achievements, most POC diagnostics for animal diseases suffer from limitations such as cost effectiveness, complexity, extended analytical time, limited number of targeted analytes, lack of field testing, and improper validation, resulting in low quality tests frequently entering the market [35].

In this context, a POC device utilizing microfluidics, photonics, and communication technologies was developed within the framework of the European Union’s H2020 SWINOSTICS (swine diseases field diagnostics toolbox) project (https://swinostics.eu accessed on 21 March 2022) for the detection of six major swine viral pathogens: porcine reproductive and respiratory syndrome virus (PRRSV); swine influenza A (SIV); porcine parvovirus (PPV); porcine circovirus 2 (PCV-2); classical swine fever virus (CSFV), and African swine fever virus (ASFV). The detection of the viral antigens was mediated by photonic integrated circuits (PICs) functionalized with polyclonal or monoclonal antibodies as molecular recognition elements (MREs) [36].

The objectives of this study were to provide the first validation data of the novel POC device for PRRSV and SIV detection in oral fluid samples and assess the performance of the device by determining key performance metrics such as the limit of detection (LOD), sensitivity, specificity, accuracy, precision, positive likelihood ratio (PLR), negative likelihood ratio (NLR), and diagnostic odds ratio (DOR).

## 2. Materials and Methods

### 2.1. Samples

PRRSV type 1 Lelystad strain, provided by Professor I. Bossis (University of Maryland, College Park, MD, USA), was propagated in sub-confluent cultures of primary alveolar macrophages (PAMs) and maintained in RPMI medium supplemented with 1% glutamine, 10% fetal bovine serum, and 1% antibiotic mixture (100× pen-strep). In a 96-well plate, 100 μL cell suspension per well were inoculated with 50 μL of 10-fold serial dilutions of PRRS positive sera (tested with reverse transcription PCR) to titrate the sample. Cytopathic effect (CPE) was observed daily. At day 2 post-inoculation, 25 μL of the supernatant were transferred to freshly seeded PAM cells and CPE was observed every day (second pass). The cells were incubated at 37 °C and in a 5% CO_2_ atmosphere. Wells in which PAM cells showed CPE at both passages were considered positive. Finally, the viral copies per mL of the supernatant were estimated by real-time reverse transcription PCR.

Swine influenza H1N1 and H3N2 field isolates (laboratory confirmation and isolation was conducted at the Department of Pathology, University of Veterinary Medicine, Budapest) from clinical cases of swine influenza in Hungary were serially propagated in 9-day old embryonated chicken eggs by inoculating 100 μL of the field sample (swabs in viral transport media) in the chorionic space of eggs. Chorioallantoic fluids were collected 72 h post-inoculation, diluted 1:10 with phosphate buffered saline (PBS) and re-inoculated on 9-day old embryonated eggs. Chorioallantoic fluids were then collected 48 h post inoculation and clarified by high-speed centrifugation. SIV particles were precipitated using 5.5% *w*/*v* PEG-6000. Total protein concentration was determined using a spectrophotometer and absorbance values at 215 and 225 nm. Viral precipitates and chorio-allantoic fluids were stored at −80 °C. The viral copies per mL of sample were estimated by real-time reverse transcription PCR.

Oral fluid samples were retrieved from four countries: Greece, Italy, Hungary, and Poland. Samples were transported to the laboratory at 4–6 °C and processed within 24 h. Oral fluids were freeze–thawed, centrifuged at 12,000× *g* for 10 min and supernatants were stored at −80 °C. PRRSV functionalized sensors were tested with 37 negative and 38 PRRS positive samples (17 spiked and 21 clinical positive samples) for the assessment of the performance of the POC device. Six serial 3-fold dilutions of the reference PRRSV type 1 sample in oral fluids were used for the estimation of the limit of detection (LOD) of the sensors. SIV functionalized sensors were tested with 17 negative and 17 SIV positive samples (15 spiked and 2 clinical positive samples). For the estimation of the limit of detection (LOD), six serial 3-fold dilutions of the reference SIV sample in the oral fluids were used. The status (negative, positive) of all samples was confirmed in the laboratory using conventional and quantitative reverse transcription PCR (Section 2.2 and Section 2.3).

### 2.2. Conventional Reverse Transcription PCR (RT-PCR) Assays

Viral PRRSV and SIV RNA were isolated using the PureLink™ Viral RNA/DNA Mini Kit (Invitrogen, Carlsbad, CA, USA). The RNA isolation protocol was performed according to the manufacturer’s instructions using a standard volume of 200 μL per sample. Nucleic acids from each sample were eluted in 20 μL of elution buffer and stored at −80 °C. Reverse transcription of total RNA to cDNA was performed with random primers using the High-Capacity cDNA Reverse Transcription Kit with RNase Inhibitor (Applied Biosystems™, Vilnius, Lithuania) and standard reaction volumes of 20 μL (10 μL sample and 10 μL kit reagents), according to the manufacturer’s instructions. The cDNA was stored at −20 °C.

PRRSV cDNA was detected with conventional PCR using three primer sets targeting the open reading frames (ORF) ORF1b and ORF7 (Table 1). SIV cDNA was detected with conventional PCR using two primer sets (SIV_Set_1–2, Table 1) targeting the M and NP genes, respectively. SIV was typed using seven additional primer sets. The primer sets and products of PRRSV and SIV amplification are presented in Table 1. All conventional PCR assays were performed in a total volume of 25 μL, consisting of 22.5 μL PCR 1.1× SuperMix (Invitrogen, Carlsbad, CA, USA), 0.5 μL of 10 μM forward primer solution, 0.5 μL of 10 μM reverse primer solution, and 1.5 μL of template cDNA. Cycling conditions were optimized for each primer set (Table 2). PCR products were analyzed in 2% agarose gel and stained with ethidium bromide. A 100 bp ladder (Thermoscientific, Vilnius, Lithuania) was used to assess the amplicon length.

### 2.3. Quantitative Reverse Transcription PCR (RT-qPCR) Assays

Quantitative PCR for the detection of viral cDNA isolated from clinical and spiked samples was performed in triplicate using SYBR Green chemistry and the primer set PRRS_Set_1 (ORF1b gene) and SIV_Set_1 (M gene) for PRRSV and SIV, respectively. The reactions were performed in a total volume of 20 μL, consisting of 10 μL 2× PowerUp™ SYBR™ Green Master Mix with 500 nm ROX (Applied Biosystems, Vilnius, Lithuania), 0.5 μL of 10 μM forward primer solution, 0.5 μL of 10 μM reverse primer solution, 1 μL of template cDNA, and 8 μL H_2_O. Cycling conditions were as follows: Initial activation of UDG for 2 min at 50 °C, activation of the Dual-Lock polymerase for 2 min at 95 °C, 40 cycles of denaturation at 95 °C for 15 s, and annealing and extension at 60 °C for 1 min. Data were collected with a 7500 Real Time PCR System and analyzed with 7500 software, v.2.0.6 (Applied Biosystems). Quantification of the viral load was performed with a standard curve generated from known amounts of gel purified and sequenced PRRSV and SIV PCR-amplified cDNA, ranging from 10^10^ to 10^3^ genomes per PCR reaction, in duplicate. Sequencing was performed to verify the identity of the PCR amplicons and accurately determine the MW of the fragment. Measurement of the DNA mass was performed using the Quawell Q5000 (Quawell, San Jose, CA, USA) spectrophotometer. PRRSV and SIV concentrations were expressed as the viral copy number per mL of sample.

### 2.4. POC Device, Sensors, and Antibodies

**Figure 1:**
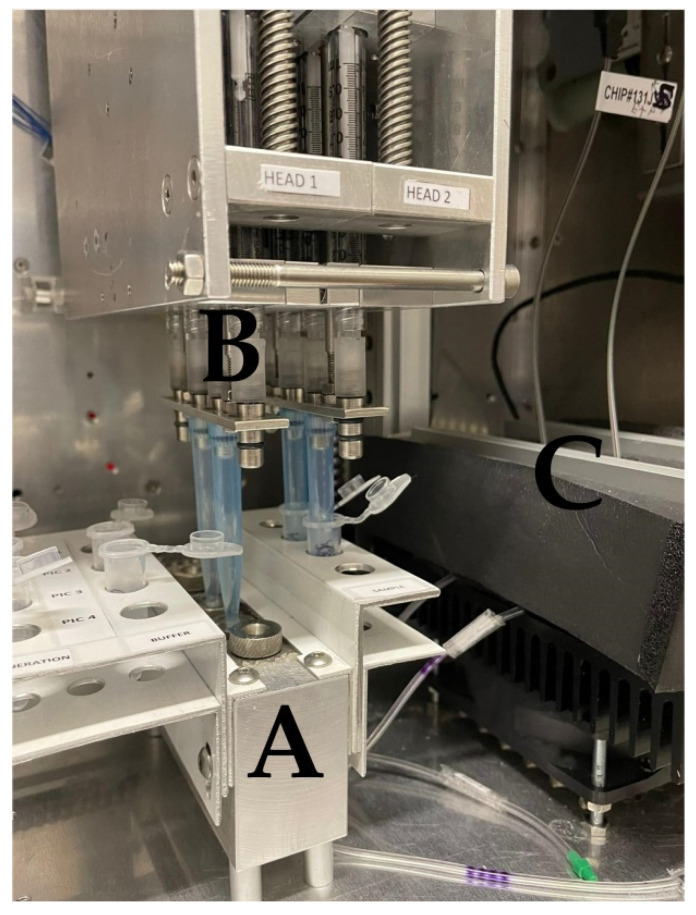
A—Sample and buffer holder. Pipette tips are pressed against the microfluidic inlet (circular button on the top area of the holder) to propel the fluids to the sensors. B—Syringe system that draws and propels fluids, C—Insulating material. Under it lie the sensors on a Peltier surface that keeps the temperature steady at 25 °C. The optic fibers (tagged cables) of the PICs exit the insulating material through holes.

The POC device has been previously described in detail [43]. In brief, the system utilizes photonic integrated circuits (PICs) on silicon nitride, coupled with a tunable laser and a photodiode. Buffers and samples are propelled by syringes to the sensors through microfluidic channels (Figure 1). A Peltier element is used to control the temperature. A microcontroller and an Arduino datalogger are used for the operation of the device and for data recording, respectively. Users can interact with the device via a mobile application interface. Data are recorded on a cloud platform that generates simple yes/no results in real time, enabling data sharing to authorized personnel/veterinarians. In the absence of an Internet connection, the results can be stored and uploaded later in the cloud platform. The assay is completed within 60 min. Minimal handling and training are required for the operation of the device and end-users only need to add pipette tips and samples in the device. The device allows for multiplexing, as a sample can be tested simultaneously for the panel of six swine viral diseases. All modules were integrated into a single device that had a size of 40 × 50 × 60 cm, weighed 45 kg, and was powered by a single plug (connected to 220 V socket). The device underwent initial field testing at commercial swine farms in Greece, Italy, and Hungary.

**Figure 2:**
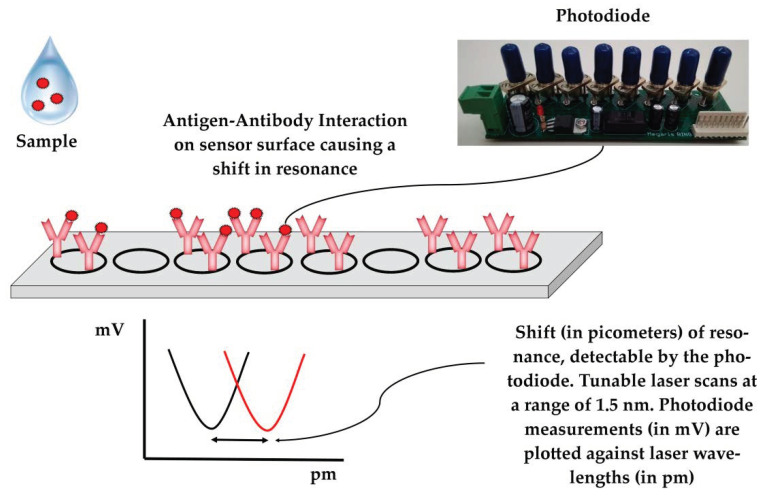
Sensors and the principle of viral detection.

A detailed description of the sensors has previously been published [44,45]. In short, photonic integrated circuits (PICs) utilize eight ring resonators split into two blocks consisting of four rings each. In each block, three rings are functionalized with immobilized antibodies for a given disease on their surfaces (e.g., PRRS or SIV) and the surface of one ring is blocked with fish gelatin and serves as the reference. Each ring is excited by the laser at a continuous wavelength in the range of approximately 1.5 nm and only resonates at a specific wavelength. The resonant wavelength remains trapped in the ring, resulting in a minimum in the wavelength spectrum, which is detected by the device’s photodiode. Antibody–antigen interaction results in a localized change in the refractive index, which extends beyond the sensor’s surface [46], shifting the resonant wavelength of the antibody-functionalized rings (Figure 2). Polyclonal anti-PRRS antibody pAb PRSNP11-S (Alpha Diagnostic, San Antonio, TX, USA), which recognizes the PRRSV type 1 nucleocapsid protein, was used to detect PRRSV in the samples. For the detection of SIV, sensor rings were functionalized with mAb MA5-17101 (Thermo Fisher Scientific, Waltham, MA, USA) monoclonal antibodies, which recognize the influenza A virus nucleoprotein. Both antibodies were selected to recognize conserved regions to facilitate the detection of a wide range of circulating viral strains.

### 2.5. Analysis Protocol and Shift Calculation

The analysis protocol was optimized for the detection of PRRSV and SIV in complex biological matrices (oral fluids). The analysis protocol for PRRSV included five consecutive steps:

1. The buffer step: The buffer used was MES 0.1 M + 1% *w*/*v* BSA, pH = 6, which flowed for 15 min at a flow rate of 30 μL/min. During this step, the signal was stabilized for the establishment of a baseline.
2. The sample step: The sample (300 μL) was diluted at a ratio of 1:1 with MES 0.1 M + 1% *w*/*v* BSA, pH = 6. The sample flowed for 10 min at a flow rate of 30 μL/min. Binding of the analytes on the functionalized PIC surfaces occurred during this step.
3. The washing step: The buffer used was MES 0.1 M + 1% *w*/*v* BSA, pH = 6, which flowed for 15 min at a flow rate of 30 μL/min. Unbound viral particles and sample residues were removed at this step.
4. The PIC surface regeneration step: The buffer used was 50 mM Glycine + 50% *v*/*v* ethylene glycol, pH = 3, which flowed for 5 min at a flow rate of 30 μL/min. During this step, PIC surfaces were regenerated by releasing the captured antigens from the antibodies.
5. The final washing step: The buffer used was MES 0.1 M, pH = 6, which flowed for 5 min at a flow rate of 30 μL/min. In this step, BSA was excluded from the washing buffer to prevent protein accumulation in the microfluidic channels of the sensors.

A similar protocol, previously described [43], was exploited for the detection of SIV, with the main difference being the use of PBS + 0.05% *v*/*v* Tween 20 + 1% *w*/*v* BSA, pH = 7.4, instead of MES 0.1 M + 1% *w*/*v* BSA, pH = 6. Outflows were delivered to a waste tank for UV sterilization.

Shift calculation was performed with a calculation algorithm written in Python, which is accessible through the mobile application software. The algorithm exploits the LOWESS algorithm [47] to produce a smoothed scatterplot of mV values (recorded by the photodiode) versus the laser wavelength values (the excitation wavelength produced by the tunable laser), allowing for shift calculation in each ring (Figure 2). The resonant shift (absolute values in pm) of reference rings (immobilized fish gelatin on their surface) is subtracted from the resonant shift (absolute values in pm) of the antibody-functionalized rings to estimate the resonant shift in pm, which is truly attributable to the antigen–antibody interactions (Shift_interaction_). Shift_interaction_ values greater than 0 are considered as positive results (i.e., detection of viruses); Shift_interaction_ values equal or less than 0 are considered as negative results (i.e., absence of viruses).

### 2.6. Limit of Detection (LOD) Experiments

Reference samples for both PRRSV and SIV were quantified using the previously described qPCR assays. To estimate the device’s limit of detection (LOD) for PRRSV, six serial 3-fold dilutions of the PRRSV reference samples in oral fluids starting from 10^8^ viral copies/mL were prepared and tested against a total of six sensors. Respectively, to estimate the device’s LOD for SIV, six serial 3-fold dilutions of the SIV reference samples in oral fluids starting from 10^7^ viral copies/mL were prepared and tested against two sensors.

### 2.7. Validation and System Performance

LOD shift values (presented in detail in the Section 3) could not fit to a linear model, leading to the adoption of a qualitative system with a binary response variable (positive, negative) for the interpretation of results. The diagnostic performance of the device was assessed by estimating its sensitivity, specificity, accuracy, precision, positive likelihood ratio (PLR), negative likelihood ratio (NLR), and diagnostic odds ratio (DOR). To estimate the numbers of True Positives (TP), True Negatives (TN), False Positives (FP), and False Negatives (FN), calibrators were classified into three categories as previously suggested [48]. Positives (P) were considered samples that had Ct vales lower than 30 in real-time PCR. Low Positives (LP) were considered as samples with Ct values equal or higher than 30. Negatives were considered as samples that tested negative with conventional and real-time PCR methods. The sensitivity (TP/(TP + FN)), specificity (TN/(FP + TN)), accuracy ((TP + TN)/(TP + TN + FP + FN)), precision (TP/(TP + FP)), PLR (sensitivity/(1 - specificity)), and NLR ((1 - sensitivity)/specificity) as well as their 95% confidence intervals (95% CI) were calculated including both the positive and low positive calibrators using MedCalc online software (https://www.medcalc.org/calc/diagnostic_test.php, accessed on 14 March 2022). Finally, DOR ((TP/FP)/(FN/TN)) was calculated to provide a global estimator, unaffected by disease prevalence, of the discriminative power of the diagnostic device, allowing for comparisons between different diagnostic tests [49]. The diagnostic odds ratio of a test is the ratio of the odds of positivity in the positive group, relative to the odds of positivity in the negative group. The positive and negative groups are defined by the golden standard method. The 95% CI of DOR was calculated by using the formula Log(DOR) ± 1.96SE(Log(DOR)), where SE(Log(DOR)) = √(1/TP + 1/TN + 1/FP + 1/FN) [49].

PRRSV samples (37 negative and 38 positive samples) were tested with a total of 20 PRRSV-functionalized PICs. SIV samples (17 negative and 17 positive) were tested with 11 SIV functionalized PICs. The PICs could be used six times without compromising their performance. Due to minor structural deterioration of the PICs, additional experiments were not attempted. Each PIC had three functionalized rings for each disease and provided multiple independent measurements for a single sample. PRRSV-functionalized rings provided 277 valid results and SIV-functionalized rings provided 100 valid results. Given that each ring functions independently, the validation of the system performance was conducted at the ring level.

### 2.8. Statistical Analysis

Mean shift values from the LOD experiments were plotted against their respective viral concentrations (in log10 (Viral copies/mL)). A normality check and an independent samples *t*-test was conducted to assess any differences in shift values between the spiked (n = 17) and clinical (n = 21) PRRSV positive samples. Receiver operating characteristic (ROC) curves were drawn and the area under the curve (AUC) and the respective 95% confidence intervals (95% CI) for both PRRSV and SIV were calculated. Test outcomes (TP, TN, FP, and FN) were calculated for each disease using the optimal threshold of the ROC curve analysis, and subsequently, the diagnostic performance of the PICs was estimated. The statistical analysis was performed with SPSS v23 software (IBM Corp., Armonk, NY, USA).

## 3. Results

### 3.1. RT-PCR and RT-qPCR Results

**Figure 3:**
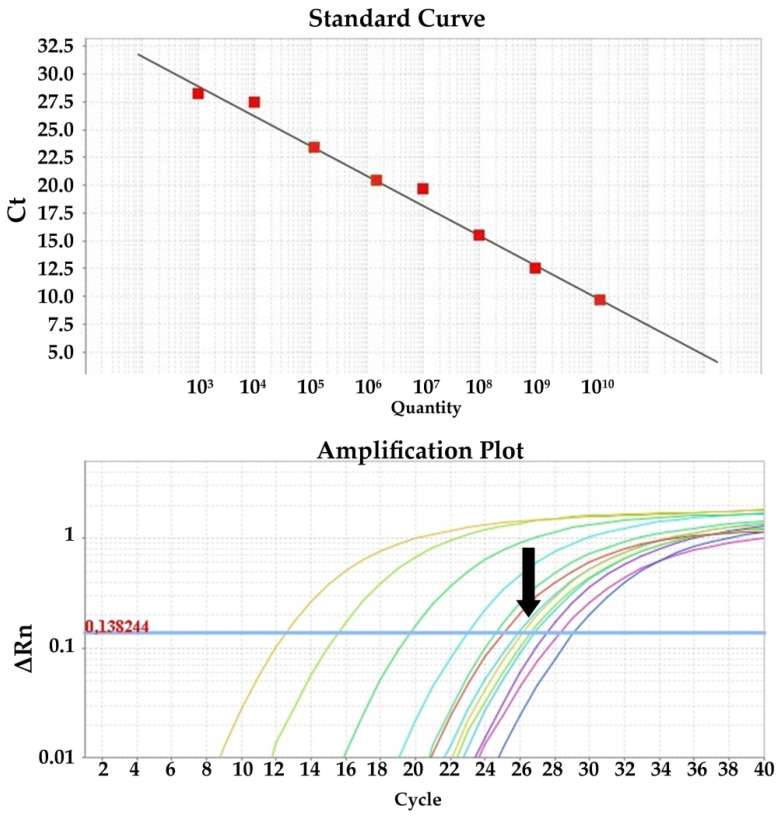
RT-qPCR standard curve using the PRRS_Set_1 (ORF1b gene) primer set and the amplification plot with field samples indicated with the black arrow.

**Figure 4:**
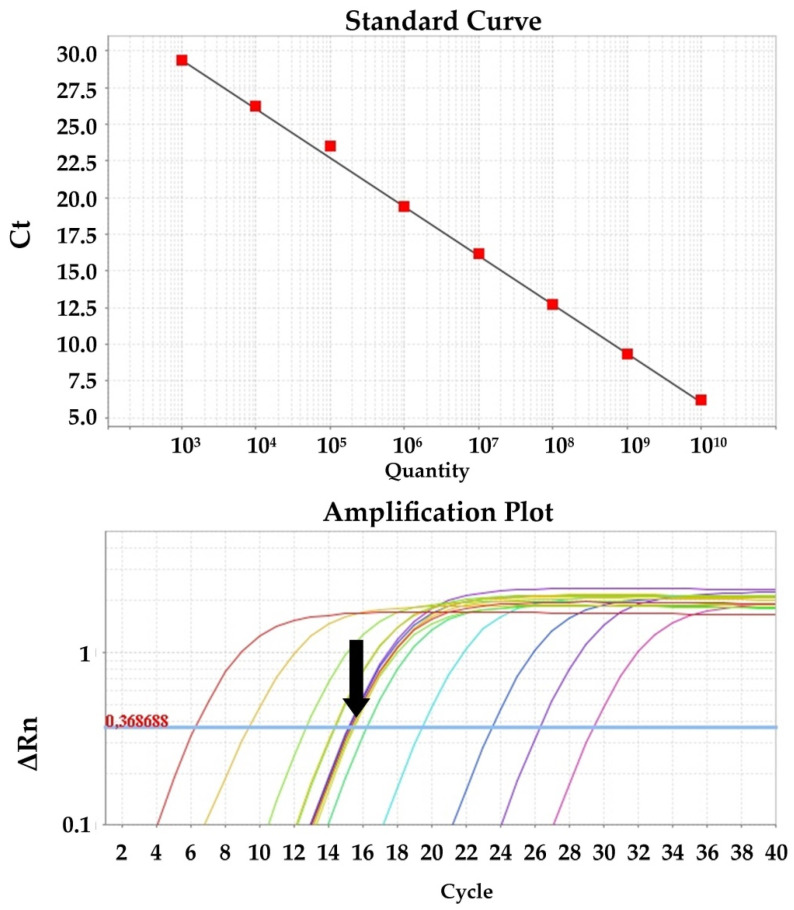
RT-qPCR standard curve using the SIV_Set_1 (M gene) primer set and the amplification plot with reference samples indicated with the black arrow.

Samples were screened with the previously mentioned RT-PCR assays. All samples included in this study met the following qualification criteria: (i) PRRSV negative samples should test negative with all three of the PRRSV-specific primer sets; (ii) PRRSV positive samples should test positive with all three of the PRRSV-specific primer sets and additionally be typed as PRRSV-1 (PRRSV_set_3); (iii) SIV negative samples should test negative with both the SIV_set_1 and SIV_set_2 primer sets; and (iv) SIV positive samples should test positive with both the SIV_set_1 and SIV_set_2 primer sets and additionally be successfully typed (SIV_set_3-9). The SIV positive samples used in this study belonged to commonly circulating H1N1 or H3N2 subtypes. All positive samples were quantified with the SYBR Green RT-qPCR assays. Samples used in the LOD experiments were also quantified (viral copies/mL of sample) with the RT-qPCR method using the standard curves produced in this study (Figure 3 and Figure 4).

### 3.2. Limit of Detection (LOD)

**Figure 5:**
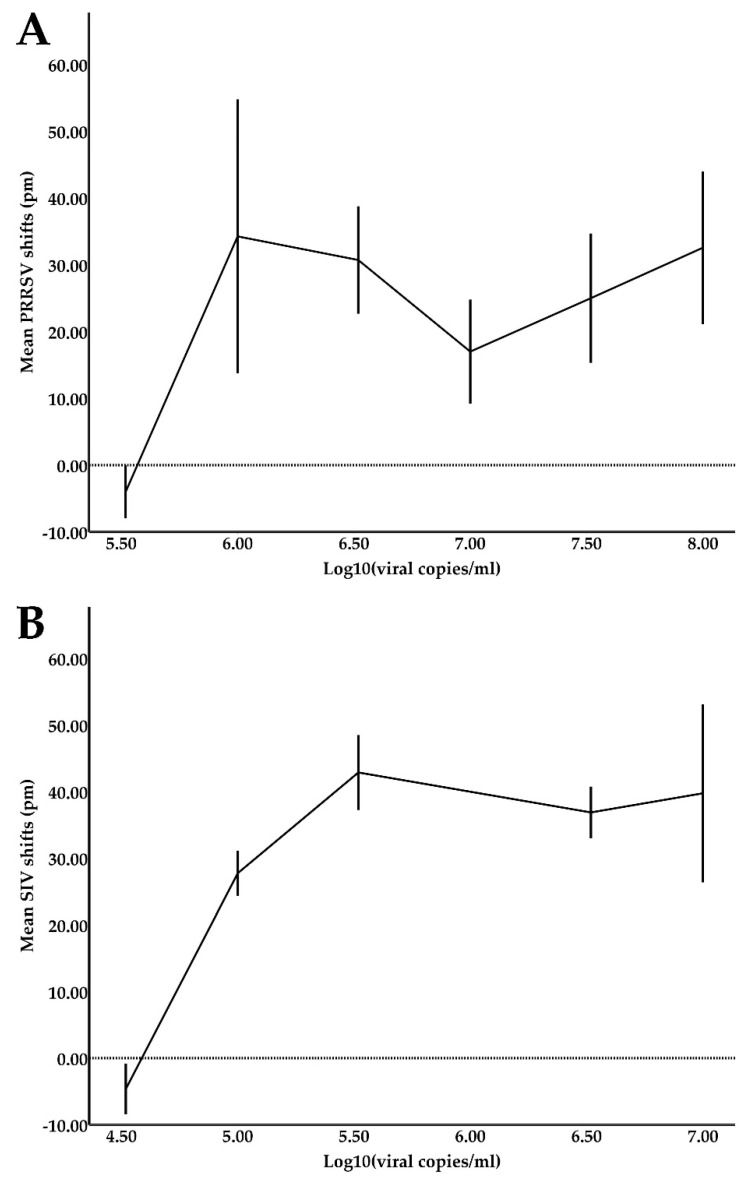
(**A**) PRRSV and (**B**) SIV shift responses (in pm) plotted against viral concentrations (Log10 (viral copies/mL)).

In the image below (Figure 5), the shift response in picometers (pm) of the PRRSV- and SIV-functionalized rings were plotted against the respective viral concentrations [Log_10_ (viral copies/mL of sample)] of the samples. The error bars represent the standard errors of the shifts in each viral concentration. Shift responses tending to zero indicate the lowest viral concentration of samples that was detectable by the sensors (i.e., the LOD).

The PRRSV- and SIV-functionalized rings showed a LOD of approximately 3.3 × 10^5^ viral copies/mL and 3.3 × 10^4^ viral copies/mL, respectively, which was determined as the lowest viral copy number that produced positive shift values. The lower LOD achieved by SIV sensors may be due to the fact that SIV sensors were produced at a later stage than the PRRSV sensors, and previous experience was exploited during their manufacture. As shown in Figure 5, shift responses in pm are not dose-dependent due to the prozone effect. This phenomenon led to the adoption of a qualitative response (yes or no) system.

### 3.3. Receiver Operating Characteristic (ROC) Curve

**Figure 6:**
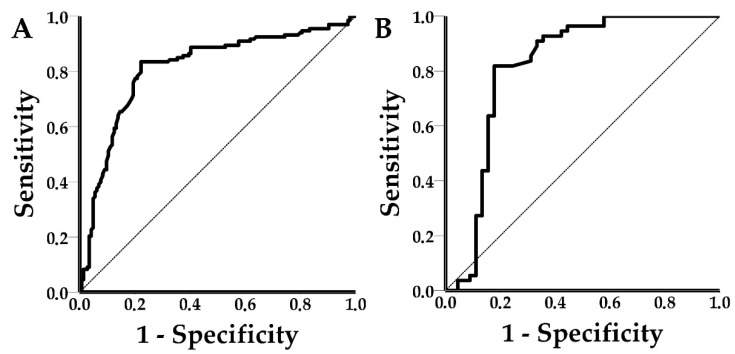
(**A**) PRRSV ROC curve, AUC: 0.812, 95% CI: 0.759–0.866, *p* < 0.0001 and (**B**) SIV ROC curve, AUC: 0.816, 95% CI: 0.719–0.912, *p* < 0.0001.

The difference in shift responses for the spiked and clinical PRRSC samples was not statistically significant (*t*-test, *p*-value = 0.067). Thus, for the estimation of the AUC, all 277 and 100 valid shift responses for PRRSV and SIV, respectively, were used. AUC represents the chances that the device will correctly distinguish the positive class values from the negative class values. In the case of PRRSV, an AUC value of 0.812 (95% CI: 0.759 to 0.866, *p* < 0.0001) and an optimal shift threshold equal to 5.5 pm (83.5% sensitivity, 77.8% specificity) were achieved (Figure 6). SIV had an AUC value of 0.816 (95% CI: 0.719 to 0.912, *p* < 0.0001) and an optimal shift threshold equal to 3 pm (81.8% sensitivity and 82.2% specificity) (Figure 6). In both cases, the optimal thresholds were selected using Youden’s index (=sensitivity + specificity − 1).

### 3.4. Validation and System Performance Results

The optimum shift thresholds calculated in the ROC curve analysis were used to classify the shift responses in the TP, TN, FP, and FN (Table 3) and consequently assess the performance of the device. The performance metrics (sensitivity, specificity, accuracy, precision, PLR, NLR, DOR), along with their 95% CI, are shown in Table 4.

## 4. Discussion

This study demonstrated that the novel POC device could successfully integrate the PICs, photonics, microfluidics, and communication technologies for the label-free detection of PRRSV and SIV in spiked and clinical oral fluid samples. The achieved LOD values for PRRSV and SIV were 3.3 × 10^5^ viral copies/mL and 3.3 × 10^4^ viral copies/mL, respectively. The diagnostic performance of the POC device was satisfactory at the ring level. PPRSV-functionalized sensors achieved a sensitivity of 83.5%, specificity of 77.8%, accuracy of 80.5%, precision of 77.6%, PLR of 3.76, NLR of 0.21, DOR of 17.66, and AUC values of 0.812. The respective values for SIV functionalized sensors were 81.8%, 82.2%, 82%, 84.9%, 4.60, 0.22, 20.81, and 0.816. At first glance, SIV-functionalized rings seemed to outperform the PRRSV-functionalized rings, however, the 95% CI of all the PRRSV and SIV metrics overlapped, and the recorded differences were not statistically significant. The wider 95% CI of the SIV metrics was due to the lower number of observations in the SIV group.

In another study, an integrated microfluidic platform developed for the multiplex detection of the anti-PRRSV, -CSFV, and -PCV-2 circulatory antibodies achieved sensitivities of 89.74%, 96.61%, and 88.89%; specificities of 96.61%, 97.22%, and 98.31%; accuracy values of 93.88%, 96.84%, and 94.74%; and AUC values of 0.968, 0.992, and 0.989, respectively, when tested with 100 serum samples [34] Nevertheless, the 95% CIs were not presented in that study and the results could not be directly compared with the results presented herein as any statistically significant differences could not be established.

In this work, the validation analysis and the calculation of the performance metrics were conducted at the ring level of the PIC. Each PIC had three rings functionalized for each targeted analyte (PRRSV or SIV). The device could achieve a much higher performance (approximately 90% sensitivity and specificity) at the test level, similar to those of other studies [34], by compiling the information retrieved by the three functionalized rings for each virus. This can be undertaken by considering the response of the majority of the rings as the valid test result. For example, suppose that two out of three rings provide a “yes” answer and one ring provides a “no” answer. In that case, the valid result at the test level should be considered as “yes” (i.e., detection of the targeted analyte). Be that as it may, this approach is far from the original aspiration, which was that a single ring would suffice for the successful detection of analytes.

Reliable POC testing can be a valuable tool to mitigate swine viral diseases and their socioeconomic impact. However, POC tests may underperform when implemented by untrained personnel outside the limits of the well-defined populations of validation studies. The novel POC device utilizes microfluidics, photonics, and information and communications technology to automatically detect PRRSV and SIV. The device is operated via a mobile application and the results are generated in a cloud-based platform. Users must only add pipette tips and samples to the device, thus minimizing the user-introduced bias in the photonic measurements. A detailed description on the use of the device has been previously published [43].

From a biological perspective, the performance of the device depends on the selection of appropriate antibodies to enhance the efficiency of the bio-recognition event on the sensor’s surface. In this work, the polyclonal pAb PRSNP11-S (Alpha Diagnostic) antibody, which recognizes the PRRSV type 1 nucleocapsid protein, and monoclonal mAb MA5-17101 (Thermo Fisher Scientific) antibody, which recognizes the influenza A virus nucleoprotein, were selected. Both antibodies were carefully chosen to recognize conserved viral proteins, which are also expressed in relatively large quantities, to allow for the detection of a wide range of circulating viral strains. The inclusion of mild detergents in the sample buffer results in partial disassembly of the virus envelope and matrix proteins, enabling the detection of antigens in the interior of the virion. The validation process for the selected antibodies has previously been described [45]. In the initial version of the PIC sensors used in this study, antibodies were not immobilized in an oriented way (binding of the antibody Fc region on the sensor surface). Despite the random immobilization, the number of antibodies used was sufficient for the bio-recognition event to take place.

The LOD values achieved in this study were satisfactory for the diagnosis of clinical cases for both PRRSV (LOD of 3.3 × 10^5^ viral copies/mL) and SIV (LOD of 3.3 × 10^4^ viral copies/mL). The samples were used directly in the device without a pre-treatment/enrichment step, except for filtering with syringe filters to remove large particulate matter that could block the microfluidic channels. In comparison, a PCR and a RT-LAMP assay, which were translated into microfluidic chips (LOC devices), achieved LOD values of 10^3^ copies/mL and 10^3^–10^4^ copies/mL, respectively [32,33]. However, both assays required a sample pre-treatment step (isolation of nucleic acids).

ROC curves were used to establish a signal threshold and consequently classify shift responses to positives or negatives. The thresholds were defined using Youden’s index to achieve the optimum performance of the device. ROC curves can be practical tools to assess the sensitivity–specificity tradeoff of diagnostic assays. Specifically, ROC curves can help to establish the desired levels of sensitivity or specificity based on disease characteristics and epidemiology. For example, in the case of POC testing for African swine fever, sensitivity (or in other words high negative predictive value) is a priority, considering the socioeconomic impact of the disease. The use of Youden’s index resulted in shift thresholds of 5.5 pm and 3 pm for PRRSV and SIV, respectively. The achieved sensitivity for PRRSV was 83.5% (95% CI: 76.03–89.33%) and for SIV 81.8% (95% CI: 69.10–90.92%). The achieved specificity for PRRSV was 77.8% (95% CI: 70.10–84.28%) and for SIV was 82.2% (95% CI: 67.95–92.00%). Although the achieved sensitivities and specificities may seem suboptimal, it is important to note that low positive calibrators (samples with low copy number and Ct values equal to or larger than 30) were included in the study to avoid the spectrum bias, which tends to overestimate the performance of the device [50]. Additionally, PCR, a highly sensitive and specific method which is currently the diagnostic “golden standard” in veterinary practice for viral diseases, was used as the reference method, thus a lower performance was expected.

Sensitivity and specificity are intrinsic test characteristics and easily understood metrics in general; however, their values may change when the test is carried out in a different setting and/or different populations and these metrics do not suffice to assess the post-test probability and interpret the test results [50]. Additionally, the assessment of the performance of a diagnostic test should concurrently take under consideration the sensitivity and specificity. To provide a more complete view on the performance of a diagnostic device, other performance metrics should also be evaluated.

Accuracy of a diagnostic test is defined as the proportion of true classifications out of all classifications and includes the estimates of pre- and post-test probabilities. PRRSV and SIV had an accuracy of 80.5% (95% CI: 75.34–85.00%) and 82% (95% CI: 73.05–88.97%). Accuracy is handy to evaluate diagnostic tests using a single metric, but is slightly affected by disease prevalence (in both the PRRSV- and SIV-functionalized rings accuracy was affected less than 1% by prevalence) and weighs the effect of false positives and false negatives equally. Consequently, comparisons of diagnostic performance solely based on accuracy can be misleading.

The novel POC device had precision values of 77.6% (95% CI: 71.69–82.62%) and 84.9% (95% CI: 74.77–91.43%) for PRRSV and SIV, respectively. The difference in precision between PRRSV and SIV was not statistically significant (overlapping 95% CIs) and the higher precision values of SIV were due to the higher prevalence (55% in the SIV group vs. 48.01% in the PRRSV group) of positives in the SIV group. Increased prevalence in the screened population increased precision (TP/(TP + FP)) by reducing FP, as less negatives were tested. Vice versa, low prevalence resulted in reduced precision (by increasing the FP). For example, two SARS-CoV-2 nucleic acid amplification tests achieved precision values of 61.8–89.8% and 20.1–73.8% in a low-prevalence (0.14–0.41%) setting [51]. Both precision and negative predictive values are highly sensitive to prevalence, making them unsuitable for the evaluation of different diagnostic tests. The lack of epidemiological data and surveillance of swine viral diseases and the differences in disease prevalence between animal groups, farms, regions, and/or countries render precision and negative predictive values impractical for the estimation of post-test probability in animal POC diagnostics.

To counter this issue, prevalence-independent diagnostic performance metrics such as PLR and NLR have been suggested. PLR and NLR are useful metrics to link the pre- and post-test probabilities of a diagnostic test by showing how many times more likely a particular test result is in the “diseased” group in comparison with the “healthy” group. A PLR greater than 1 indicates that the test result is associated with the presence of the disease and an NLR of less than 1 indicates that the test result is associated with the absence of the disease [52]. Likelihood ratios above 10 or less than 0.1 are considered as sufficient to rule in or rule out a disease, respectively [52]. PRRSV had PLR and NLR values of 3.76 (95% CI: 2.74–5.15) and 0.21 (95% CI: 0.14–0.31), respectively. SIV had PLR and NLR values of 4.60 (95% CI: 2.43–8.73) and 0.22 (95% CI: 0.12–0.39), respectively. Although the achieved PLR and NLR values for both viruses were not perfect, the 95% CIs did not include the value of 1, making the findings statistically significant.

DOR is unlikely to be a test-specific constant, nevertheless, it is a global measure of the diagnostic performance, which can be useful in comparisons of tests across populations regardless of the disease prevalence and is also suitable for meta-analyses [49]. DOR values range from 0 to infinity. Values lower than 1 suggest improper test interpretation, a value of 1 means that the test is not able to discriminate the diseased and healthy groups, and values higher than 1 indicate a better test performance. The novel POC device achieved DOR values of 17.66 (95% CI 13.98–21.64) and 20.81 (95% CI 10.66–30.96) for PRRSV and SIV, respectively. For both viruses, DOR values were statistically significant higher than 1, indicating that the POC device could successfully discriminate positives from negatives. Despite the usefulness of DOR, the metric depends on the spectrum of the disease (low positives), is not defined in 2 × 2 tables that contain zeros, and two tests with identical DOR can have different sensitivity and specificity [49].

At this point, it is important to underline that the calculation of the different metrics of diagnostic performance is required to provide a full view of a diagnostic test’s utility. Validation studies of POC devices should also provide the framework of decision making after interpreting the test results. To do so, a detailed description of the disease and its clinical manifestation, epidemiological data, and surveillance systems are required, although acquisition of this information is rather expensive and time consuming. This often results in POC tests successfully validated in the laboratory being rendered ineffective in practice, as the proper tool is not used in the proper framework and setting. For example, a test with 95% sensitivity and 95% specificity would achieve values of a positive predictive value, which is the most useful performance metric for veterinary medicine practitioners, of only 50% for a disease with 5% prevalence in the population. This means that the hypothetical test is not appropriate to rule in the disease, given a positive result.

Most POC tests do not undergo sufficient validation with field trials and clinical utility evaluation, thus giving little financial incentive for their commercial exploitation due to the high risk of failure of novel diagnostic tests [53]. This phenomenon is exaggerated in animal production, as the slim profit margin of both farmers, and consequently commercial companies, does not provide the necessary financial incentives for the research and development of novel POC devices.

Considering the above, we suggest that the validation of novel POC diagnostics for animal diseases should focus on three points; (i) Proof of concept experiments with reference samples; (ii) extensive laboratory testing with negative and positive samples that represent the whole spectrum of the disease for the calculation of the performance metrics of the device; and (iii) field testing to investigate the utility of the device for stakeholders. The novel POC device underwent limited field testing, nonetheless, this work focuses on the proof-of-concept of the novel POC system and the first laboratory experiments with complex sample matrices (oral fluids).

Overall, the POC device is a promising tool for the sensitive and specific detection of swine viral pathogens. Over the last years, there has been an increased research interest in photonics for the detection of pathogens and other analytes. To our knowledge, this novel POC tool is the first attempt to detect viral pathogens using PICs. PICs and emerging technologies such as advanced materials, communications, microfluidics, and microfabrication can be integrated into user-centered, compact, lab-on-a-chip devices, allowing for the translation of laboratory techniques into POC devices for field use. Microfabrication facilitates the production of sensors at the μm or even the nm scale for the transduction of interactions of biomolecules into measurable signals. PICs fabricated with this technology are ultra-sensitive to refractive index changes, providing a robust platform for the detection of swine diseases.

The POC device introduces, for the first time, the previously mentioned cutting-edge technologies into POC diagnostics, paving the way for future applications and devices. This system can reduce screening costs and minimize the time required for the diagnosis of viral diseases. More specifically, current cost estimates are EUR 0.60 per sample and the device can simultaneously analyze up to four samples within approximately 1 h. The device can be utilized at farms for the diagnosis of PRRSV and SIV to evaluate the health status of animals, slightly prior to the onset of disease given the acceptable LOD values, and support evidence-based disease control strategies. Moreover, the device could be exploited for screening purposes at border checkpoints or during the purchase of animals.

The device uses oral fluid samples for the detection of PRRSV and SIV. Oral fluids are non-intrusive samples, easy to collect, suitable for herd screening, and cost effective [54,55,56]. Following adaptions in the analysis protocol such as sample pre-treatment, different dilution factors, and the use of alternative buffers, the device can be used with alternative samples such as blood sera, swabs, and feces. The use of antibodies that recognize other antigens (viruses, circulatory antibodies, etc.) would widen the panel of analytes that could be detected with the novel POC device and enable further multiplexing.

Although the presented results are promising, the device failed to quantify samples, as clearly presented in the LOD experiments. Quantification could be greatly improved by increasing the PIC uniformity and immobilizing antibodies on sensor surfaces in an oriented way. In a previous validation study for PPV and PCV-2 using the same device, the PPV and PCV-2 AUC values could be improved from 0.820 and 0.742 to 0.892 and 0.788, respectively, just by excluding a low performing PIC [43]. Another limitation of the study is that the 95% CI for each performance metric, especially for SIV, was relatively wide. Increasing the number of tested samples could reduce the 95% CIs and provide a much more precise view of the performance of the device.

Future research should focus on three key aspects: MREs, PICs, and increasing the tested sample size. MREs that recognize different antigen epitopes could be used to identify different viral strains, viruses, and other analytes to both widen the panel of detectable diseases and improve the performance of the device. Epidemiological surveillance is required to constantly employ antibodies that recognize the majority of circulating viral strains. Furthermore, antibodies should be immobilized on sensors in an oriented way to improve the performance of the device and its quantification capabilities, whereas the immobilization of both the antibodies (for functionalized rings) and the blocking proteins (for reference rings) using 3D microprinters can reduce the signal background and refine the signal resolution. The tolerances in the manufacturing of PICs can be reduced by the standardization of materials and procedures and the completely automated PIC fabrication. Future studies should include more sample types and an increased number of tests to reduce the level of uncertainty and unveil any system/sensor limitations that were not detected in this study. Finally, extensive field validation is required to increase the technology readiness level (TRL) of the device and successfully translate the research results into a commercial POC device.

## 5. Conclusions

POC diagnostic devices can potentially reduce the time and costs required for the diagnosis of swine viral diseases, and at the same time enable rapid and local decision making for the implementation of evidence-based disease control measures. The novel device presented in this study is a promising tool with satisfactory performance that integrates photonics, microfluidics, and information and communications technology into a single, portable device, paving the way for the next generation of animal POC diagnostics. Future research will reduce the current system limitations and increase the TRL of the device for successful commercialization. The use of the device for other diseases (multiplexing) as well can contribute to the further optimization of livestock biosecurity.

## Figures and Tables

**Table 1:** Primer sets used in conventional PCR for the detection of PRRSV and SIV.

Primer Set	Target Region	Primer Sequence (5′-3′)	Amplicon Length (bp)	Reference
PRRS_Set_1	ORF1b	Forward: CCTCCTGTATGAACTTGC Reverse: AGGTCCTCGAACTTGAGCTG	Type 1 & Type 2 255 bp	[37]
PRRS_Set_2	ORF7	Forward: CCAGCCAGTCAATCARCTGTG Reverse: GCGAATCAGGCGCACWGTATG	Type 1 & Type 2 300 bp	[38]
PRRS_Set_3	ORF7	Forward: ATGGCCAGCCAGTCAATCA Reverse: TCGCCCTAATTGAATAGGTGA	Type 1 398 bp Type 2 433 bp (Discriminative primer set)	[39]
SIV_Set_1	M gene (pan-influenza A)	Forward: GACCRATCCTGTCACCTCTGAC Reverse: AGGGCATTYTGGACAAAKCGTCTA	106 bp	[40]
SIV_Set_2	NP gene (swine influenza)	Forward: GCACGGTCAGCACTTATYCTRAG Reverse: GTGRGCTGGGTTTTCATTTGGTC	200 bp	[41]
SIV_Set_3	H1 swine type hemagglutinin	Forward: GTGCTATAAACACCAGCCTYCCA Reverse: CGGGATATTCCTTAATCCTGTRGC	116 bp	[41]
SIV_Set_4	H3 swine type hemagglutinin	Forward: CTTGATGGRGMAAAYTGCACA Reverse: GGCACATCATAWGGGTAACA	133 bp	[42]
SIV_Set_5	H1 avian type hemagglutinin	Forward: GAAGGRGGATGGACAGGAATGA Reverse: CAATTAHTGARTTCACTTTGTTGC	139 bp	[42]
SIV_Set_6	H1 pandemic hemagglutinin (Pandemic 2009)	Forward: GGGCATTCACCATCCATCTACT Reverse: CCTCACTTTGGGTCTTATTGCTATTT	133 bp	[42]
SIV_Set_7	N1 swine type neuraminidase	Forward: AGRCCTTGYTTCTGGGTTGA Reverse: ACCGTCTGGCCAAGACCA	126 bp	[42]
SIV_Set_8	N2 swine type neuraminidase	Forward: AGTCTGGTGGACYTCAAAYAG Reverse: TTGCGAAAGCTTATATAGVCATGA	116 bp	[42]
SIV_Set_9	N1 pandemic hemagglutinin (Pandemic 2009)	Forward: GGGACAGACAATAACTTCTCAATAAAGC Reverse: TTCAGCATCCAGAACTAACAGGGT	100 bp	[42]

**Table 2:** Cycling conditions for each primer set.

Primer Set	Pre-Denaturation at 95 °C	Cycles	Denaturation at 94 °C	Annealing for 30 s at	Extension at 72 °C	Final Extension at 72 °C
PRRS_Set_1	2 min	32	20 s	59 °C	40 s	1 min
PRRS_Set_2	2 min	32	20 s	62 °C	40 s	1 min
PRRS_Set_3	2 min	32	20 s	57 °C	45 s	1 min
SIV_Set_1	2 min	32	20 s	63 °C	30 s	1 min
SIV_Set_2	2 min	32	20 s	63 °C	40 s	1 min
SIV_Set_3	2 min	32	20 s	63 °C	30 s	1 min
SIV_Set_4	2 min	32	20 s	56 °C	30 s	1 min
SIV_Set_5	2 min	32	20 s	57 °C	30 s	1 min
SIV_Set_6	2 min	32	20 s	62 °C	30 s	1 min
SIV_Set_7	2 min	32	20 s	57 °C	30 s	1 min
SIV_Set_8	2 min	32	20 s	58 °C	30 s	1 min
SIV_Set_9	2 min	32	20 s	64 °C	30 s	1 min

**Table 3:** The screening results for PRRSV and SIV obtained with the novel POC device versus the RT-PCR results. The number of TP, TN, FP, and FN for each sensor type are presented.

**PRRSV—Samples Status (RT-PCR)**				
Screening results obtained with the novel device		Positives	Negatives	Total
	Positives	111 (TP)	32 (FP)	143
	Negatives	22 (FN)	112 (TN)	134
	Total	133	144	277
**SIV—Sample Status (RT-PCR)**				
Screening results obtained with the novel device		Positives	Negatives	Total
	Positives	45 (TP)	8 (FP)	53
	Negatives	10 (FN)	37 (TN)	47
	Total	55	45	100

**Table 4:** Performance metrics of the novel POC device for the PRRSV and SIV functionalized sensors.

Performance Metrics	PRRSV		SIV	
	Value	95% CI	Value	95% CI
Sensitivity	83.5%	76.03–89.33	81.8%	69.10–90.92
Specificity	77.8%	70.10–84.28	82.2%	67.95–92.00
Accuracy ^1^	80.5%	75.34–85.00	82%	73.05–88.97
Precision ^1^	77.6%	71.69–82.62	84.9%	74.77–91.43
Positive likelihood ratio	3.76	2.74–5.15	4.60	2.43–8.73
Negative likelihood ratio	0.21	0.14–0.31	0.22	0.12–0.39
Diagnostic odds ratio	17.66	13.98–21.64	20.81	10.66–30.96

^1^ Accuracy and precision values are affected by the prevalence of each disease.

## Data Availability

The data presented in this study are available within the article. Raw data supporting this study are available from the corresponding author upon reasonable request.
